# Supplementary material for: Kink far below the Fermi level reveals new electron-magnon scattering channel in Fe
Source: Nat Commun. 2019 Jan 31;10:505. doi: 10.1038/s41467-019-08445-1 (PMC6355843; doi:10.1038/s41467-019-08445-1)
Supplement: Supplementary file 1 — Supplementary Information [file 41467_2019_8445_MOESM1_ESM.pdf]

Supplementary information
Kink far below the Fermi level reveals new electron-magnon scattering channel in Fe

Młyńczak et al.

Supplementary Note 1. Photoemisson matrix elements

To get access to the bulk electronic structure of Fe, we used a thin Fe film (38ML) deposited on a Au(001) single crystal. The measurements were performed at the NanoESCA beamline of Elettra, the Italian synchrotron radiation facility, using a modified FOCUS NanoESCA photoemission electron microscope (PEEM). This set-up, operating in reciprocal space mode, preserves the angular distribution of the photoemitted electrons, allowing to acquire, during a single acquisition, two-dimensional momentum maps, with a wide reciprocal space range: $k_x, k_y \in (-2, 2)$ Å^{-1} [Supplementary Figure 1(a) and (b) for s- and p-polarized light, respectively]. A photon energy of $h\nu = 70$ eV was used. The photon incidence direction is marked in Supplementary Figure 1b by a wiggly arrow. The films were remanently magnetized before each measurement, and the magnetization direction is represented by a gray arrow in the upper right corner of Supplementary Figures 1(a-b). By varying the kinetic energy of the photoemitted electrons collected into the microscope, it is possible to acquire series of patterns corresponding to different binding energies. Accordingly, the band dispersion along a chosen direction in k-space can be obtained by taking the corresponding intensity profiles within such image stacks [Supplementary Figure 1(c, d) and (e, f) for s- and p-polarized light, respectively]. On top of the experimental dispersions, results of the GW calculations are superimposed with the labels that identify the symmetry of the orbital part of the wavefunctions. Blue lines correspond to minority spin, red lines to majority spin. The identification of the experimentally observed electronic states is possible based on the consideration of the dipole selection rules, which depend on the photon polarization. For example, in case of s-polarized light and emission in the plane of light incidence [Supplementary Figure 1(d)], matrix elements favor transitions from the initial states of odd symmetry, *i.e.*, Δ_5 and $\Delta_{2'}$, and quench transitions from the initial states of even symmetry Δ_1 and Δ_2. When we examine the initial states visible in the experiment along the same direction, but excited with p-polarized light [Supplementary Figure 1(f)], we see that transitions from the states of even symmetry are allowed. This analysis enables us to identify a minority band of Δ_2 symmetry, clearly visible in dispersions measured along $k_y = 0$ with s-polarized light [Supplementary Figure 1(c)] and along $k_x = 0$ with p-polarized light [Supplementary Figure 1(f)].

Supplementary Note 2. Spin-sensitive measurement

In addition, we obtained direct information on the spin polarization of the states of interest, thanks to the result of spin-resolved measurements (Supplementary Figure 2). The spin-resolved constant-energy cut near the Fermi level measured with s-polarized light

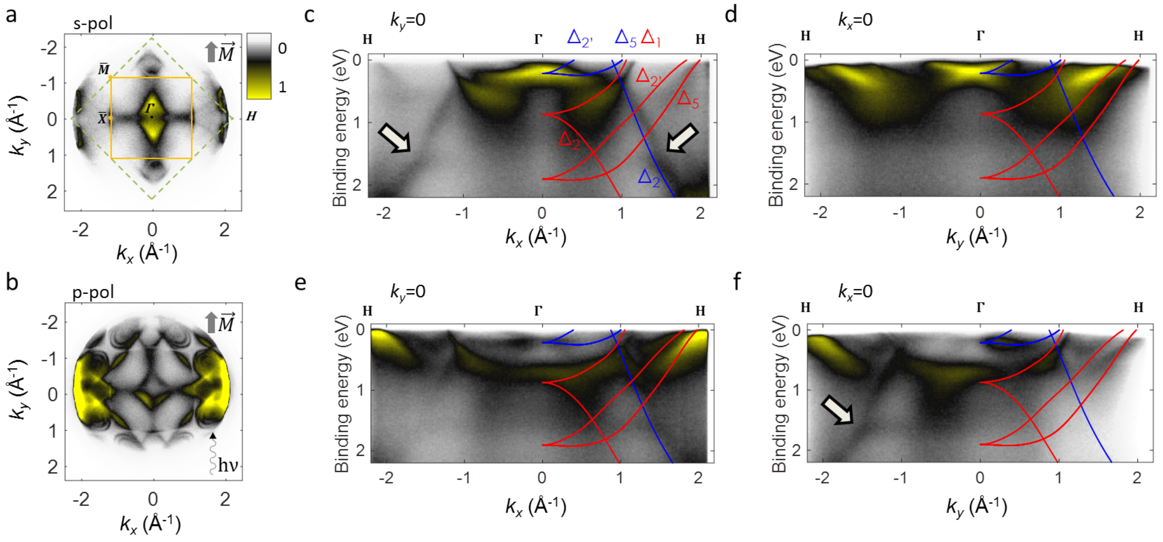

Supplementary Figure 1. Results of the measurements performed using $h\nu = 70$ eV and s- and p-polarized light. Panels (a) and (b) show constant-energy cuts near the Fermi level with squares in (a) indicating the size of the surface Brillouin zone (yellow solid line) and the edge of the bulk Brillouin zone (green dashed line). The magnetization direction is indicated by the solid arrow, a wiggly arrow in (b) indicates the photon incidence direction. Panels (c) and (e) show the dispersions along the horizontal high symmetry line of (a) and (b), respectively ($k_y = 0$), while panels (d) and (f) show the dispersions along the vertical high symmetry line of (a) and (b), respectively ($k_x = 0$). In panels (c)-(f), the results of the bulk GW calculations are included as blue and red lines for the minority and majority spin, respectively. The labels in (c) indicate the symmetry of the orbital part of the wavefunctions.

is shown in Supplementary Figure 2(a). The applied two-dimensional color map is included in the inset. The blue (red) color corresponds to minority (majority) states. The light incidence direction is marked with a wiggly arrow. Figures 2(b) and (c) present energy dispersions measured for s-polarized light. On top of the experimental dispersions, results of the GW calculations are superimposed (the same as in Supplementary Figure 1). Based on the result shown in Supplementary Figure 2(b) (Γ-H direction, for which $k_y = 0$) we can confirm the identification of a minority band Δ_2 as well as a majority band Δ_1. The high spectral intensity related to the minority bands Δ_5 and $\Delta_{2'}$ is also clearly visible. In Supplementary Figure 2(c) (Γ-H direction, for which $k_x = 0$), additional intensity from the majority $\Delta_{2'}$ band is visible.

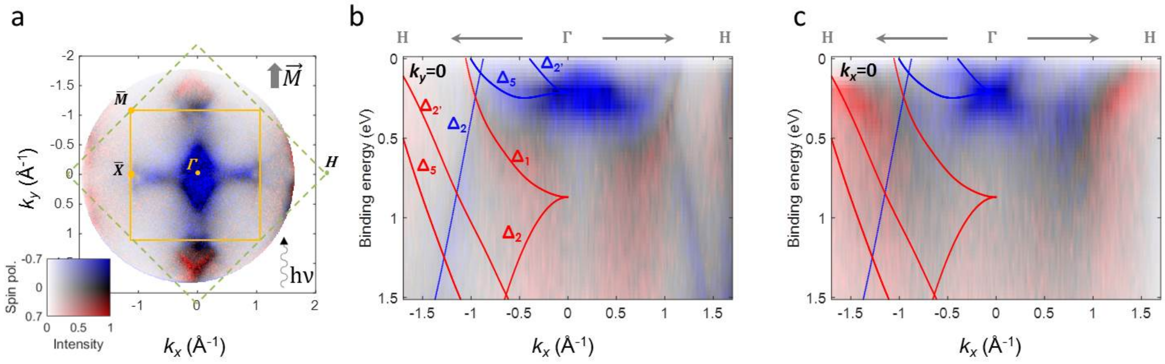

Supplementary Figure 2. Same as Supplementary Figure 1 [(a)(c)(d)] for the spin-polarized measurements.

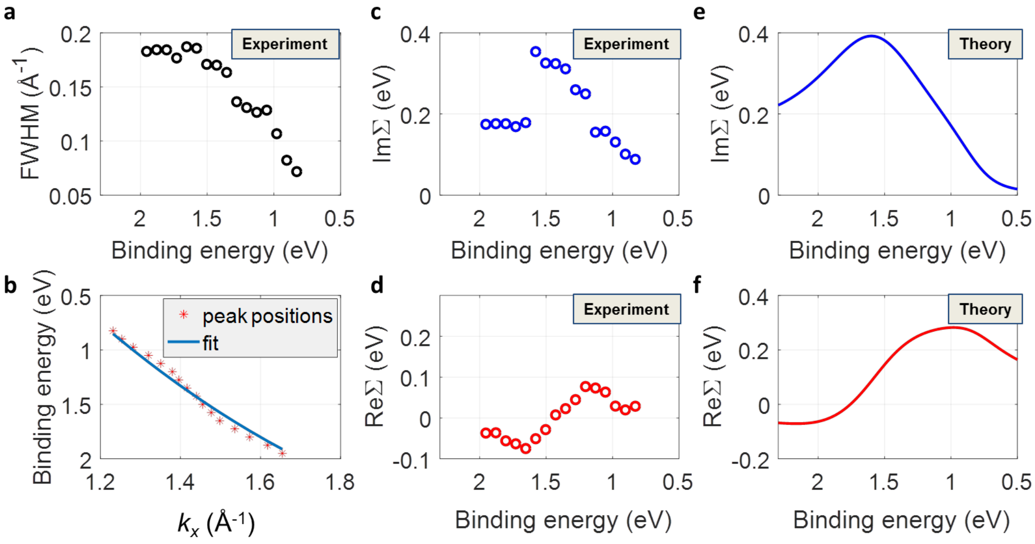

Supplementary Figure 3. Experimental and theoretical self-energy. (a) Full width at half maximum (FWHM) of the Lorentzian peaks as a function of binding energy. (b) Positions of the Lorentzian peaks that reproduce the shape of the Δ_2 band (red symbols) with a quadratic fit (blue solid line) of the GW band. (c-f) Imaginary and real parts of the self-energy determined from (c,d) experiment and (e,f) theory. See the text for details.

Supplementary Note 3. Self-energy

As a result of the interactions and correlations among the electrons in a solid, a spectral function measured by photoemission differs from the one obtained for 'undressed' particles. The quantity that embeds a single particle (electron or hole) into the interacting many-body system is the self-energy $\Sigma(\mathbf{r}, \mathbf{r}'; \omega)$, which is complex-valued: its real part describes shifts

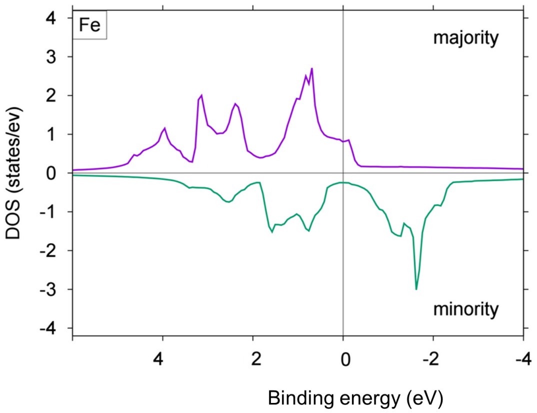

Supplementary Figure 4. LSDA density of states (DOS) of bcc Fe plotted separately for minority and majority states. The maximum of the majority DOS is found for $E_{\mathrm{b}} = 0.8$ eV.

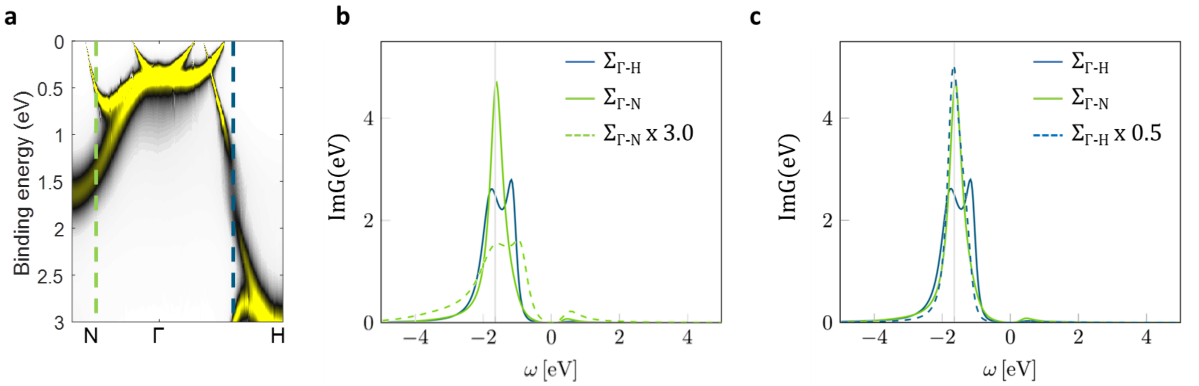

Supplementary Figure 5. Comparison to Γ-N direction. (a) Results of the *GT* calculations along Γ-H and Γ-N directions (only minority bands are shown). The band along the Γ-H direction shows a kink, the other does not. Green and blue vertical lines mark the momenta at which the spectral functions presented in (b) and (c) are taken. (b) Spectral functions along Γ-H and Γ-N directions (blue and green solid lines, respectively). A double-peak feature appears if the band dispersion exhibits a kink. When the self-energy along the Γ-N direction is scaled by three (green dashed line), a double peak develops also along Γ-N. (c) Similar to (b), but the self-energy along Γ-H is scaled by 0.5 (dashed blue line), resulting in a single peak, and as a consequence the kink disappears.

in the electron binding energy, and its imaginary part yields the lifetime broadening of the states.

The theoretical spin-σ spectral function of the interacting many-body system reads $A^{\sigma}(\mathbf{k},\omega) = \mathrm{tr}\ Im[\omega I - H^{\sigma}(\mathbf{k}) - \sigma\Delta_{\mathrm{x}} I - \Sigma^{\sigma}(\mathbf{k},\omega)]^{-1}$, where the trace is taken over all states. The quantities $\Sigma^{\sigma}(\mathbf{k},\omega)$ and $H(\mathbf{k})$ are matrices represented in terms of an orthogo-

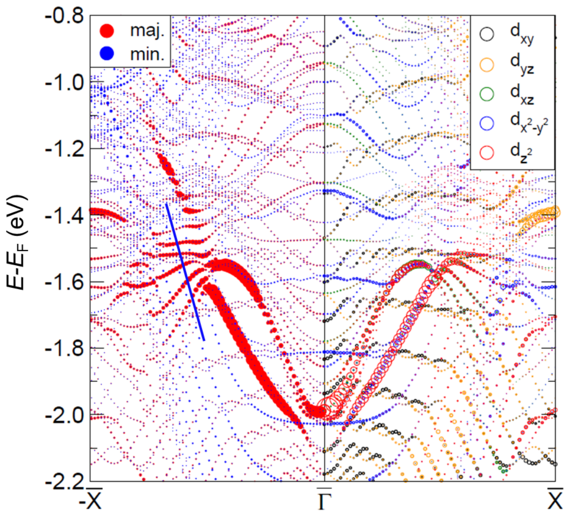

Supplementary Figure 6. Results of the relativistic DFT slab calculations of the surface electronic structure of Fe(001). The energy range where the kink was experimentally observed is shown. Left (right)-hand side of the plot shows spin (orbital) character of the surface states. On the left side, predominantly minority (majority) states are shown in blue (red). The straight blue solid line is a guide to the eye that indicates the discussed bulk band (see text). The size of the symbol corresponds to the localization of each state in the surface layer.

nal set of Bloch functions of momentum $\mathbf{k}$ (we use the LSDA eigenfunctions) with the LSDA Hamiltonian H and the identity matrix I. The parameter Δ_x is determined in such a way that the Goldstone condition is fulfilled [1–3].

The GT self-energy diagrams are shown in Fig. 1(c) in the main part of the article. From iterating the Hedin equations it follows that the lowest-order diagram is of third order in the screened interaction W, for which we use the random-phase approximation. The Bethe-Salpeter equation for the T matrix is formulated in such a way that it yields the ladder diagrams to all orders starting from the third-order diagram. Summing to all orders is necessary to obtain well-defined collective spin excitations and realistic renormalization effects in the Stoner continuum. The self-energy is calculated on an imaginary-frequency mesh and then analytically continued to the full complex plane using the Padé approximation.

The theoretical self-energy can be compared to the experimental self-energy, whose imaginary part is defined as $2\text{Im}\Sigma = \delta E = (dE/dk)\delta k$ [4], where δE and δk are the band broadenings in terms of energy and momentum, respectively. In order to obtain the self-energy from the experimental result, we fitted momentum distribution curves (MDC) with Lorentzians on a linear background (as shown in Fig. 2 in the main part of the article), extracting peak positions and full-width half-maximum (FWHM) line widths. We used the

FWHM of the fitted Lorentzians [shown as a function of binding energy in Supplementary Figure 3(a)] to find δk while dE/dk was obtained as slopes of the linear functions fitted to the fragments of the experimental dispersion. To obtain $\mathrm{Re}\Sigma(\omega)$, it is necessary to quantitatively compare the experimental dispersion with the shape of the 'undressed' band. To reproduce the shape of the theoretical band, we fitted a quadratic function to the Δ_2 band obtained from a GW calculation. Next, we used the same fit parameters, only allowing the change in the binding energy (which accounts for the possible difference in the Fermi level position between theory and experiment), to fit the experimental points [Supplementary Figure 3(b), red points], obtaining ϵ_k. The result of the fit is presented in Supplementary Figure 3(b) with a blue solid line. The real part of the self-energy is then defined as $E_k - \epsilon_k$, where E_k is the experimental dispersion. The resulting experimental $\mathrm{Im}\Sigma(\omega)$ and $\mathrm{Re}\Sigma(\omega)$ are presented in Supplementary Figure 3(c) and (d), respectively. They compare remarkably well with the corresponding calculated self-energies [Supplementary Figure 3(e) and (f)]. Importantly, $\mathrm{Re}\Sigma(\omega)$ exhibits a strong variation with binding energy and changes sign when passing the point of $E_\mathrm{b} = 1.5$ eV, where $\mathrm{Im}\Sigma(\omega)$ shows a peak. This indicates a resonance of the self-energy, which stems from the strong electron-magnon coupling.

Supplementary Note 4. Density of states

The energy of the many-body state that is responsible for the appearance of the kink is approximately given by the sum of the spin excitation (magnon) energy and the binding energies of the majority hole states that couple to the magnon. Figure 4 shows the LSDA density of states (DOS) of bulk Fe. Majority and minority DOS are shown separately. The maximum of the majority DOS is found close to $E_b = 0.8$ eV. By analyzing the theoretical data, we have revealed that the majority bands forming this DOS peak are indeed the ones that couple most strongly to the many-body spin excitation.

Supplementary Note 5. Comparison to Γ-N direction

In order to check whether the high-energy kink is a general phenomenon that would appear for all minority bands, we examined a minority band along the Γ-N direction [Supplementary Figure 5(a)]. Apparently, a high-energy kink along the Γ-N direction is not visible. Figures 5(b) and (c) show as solid lines the spectral functions taken at the momenta where the bands cross $E_\mathrm{b} = 1.5$ eV, marked by vertical lines in (a). The line corresponding to Γ-N shows a single peak, whereas the one corresponding to Γ-H shows a double peak, indicative of the kink. We have included artificial spectral functions as dashed lines, which

are obtained with self-energies scaled by factors of 3.0 (b) and 0.5 (c) for the two **k** directions, respectively. As a result of this scaling, the single peak turns into a double peak and vice-versa, which demonstrates that the appearance of the high-energy kink is highly sensitive to the strength of the coupling between the minority hole and the many-body state, which depends on the particular band and the direction in **k** space.

Supplementary Note 6. Surface electronic structure

In order to unambiguously prove that the discussed kink is not related to an anticrossing with the surface state, we have performed slab calculations to analyze in detail the spin and orbital character of the Fe(001) surface state which lies within the binding energy range of interest. The calculations were performed using density functional theory in the generalized gradient approximation [5] including spin-orbit coupling. We used 27 layers of Fe(001) and relaxed the outermost four layers using the film version of the FLEUR code [6]. The results of the calculations of the surface electronic structure are presented in Supplementary Figure 6. The left hand side of the plot shows the spin character of the surface states. The size of the symbol corresponds to the localization of the spin density of each state in the surface layer, therefore prominent red lines indicate the majority surface state. The bulk Δ_2 electronic state can be identified as one of the blue lines dispersing downwards (towards higher binding energy) from the -$\overline{\mathrm{X}}$ point towards the $\overline{\Gamma}$ point (marked by an adjacent straight blue solid line which serves as guide to the eye). Note that the bulk bands experience back-folding at the border of the surface Brillouin zone. We do not observe any signatures of hybridization between the bulk band in question and the surface state. The reason for that is the fact that the two bands have opposite spin character, which means that they could mix only due to spin-orbit coupling. We see, however, that the orbital character of the discussed states [$d_{z^2}/d_{x^2-y^2}$ in case of the Δ_2 bulk band and d_{z^2} in case of the surface state (Supplementary Figure 6, right)] results in vanishing spin-orbit matrix element, [7] which does not allow for hybridization between them.

Supplementary References

[1] Müller, M. C. T. D., Blügel, S. & Friedrich, C. Electron-magnon scattering in elementary ferromagnets from first principles: lifetime broadening and kinks. *ArXiv e-prints* (2018). 1809.02395.

[2] Müller, M. C. T. D., Friedrich, C. & Blügel, S. Acoustic magnons in the long-wavelength limit: Investigating the goldstone violation in many-body perturbation theory. *Phys. Rev. B* **94**, 064433 (2016).

[3] Müller, M. C. T. D. *Spin-wave excitations and electron-magnon scattering in elementary ferromagnets from ab initio many-body perturbation theory.* Ph.D. thesis, RWTH Aachen, Forschungszentrum Jülich GmbH, 52425 Jülich, Germany (2017).

[4] Cui, X. *et al.* High-resolution angle-resolved photoemission study of Fe (1 1 0). *Physica B: Condensed Matter* **383**, 146–147 (2006).

[5] Perdew, J. P., Burke, K. & Ernzerhof, M. Generalized gradient approximation made simple. *Phys. Rev. Lett.* **77**, 3865–3868 (1996).

[6] `http://www.flapw.de`.

[7] Konschuh, S., Gmitra, M. & Fabian, J. Tight-binding theory of the spin-orbit coupling in graphene. *Phys. Rev. B* **82**, 245412 (2010).
